# Trends in Incidence of Gastrointestinal Tract Cancers in Western Iran, 1993-2007

**Published:** 2011-11-01

**Authors:** F Najafi, H R Mozaffari, M Karami, B Izadi, R Tavvafzadeh, Y Pasdar

**Affiliations:** 1 Department of Epidemiology, Kermanshah University of Medical Sciences, Kermanshah, Iran; 2 Department of Oral Diseases, Kermanshah University of Medical Sciences, Kermanshah, Iran; 3 Department of Pathology, Kermanshah University of Medical Sciences, Kermanshah, Iran; 4 Department of Gastroenterology, Kermanshah University of Medical Sciences, Kermanshah, Iran; 5 Department of Nutrition, Kermanshah University of Medical Sciences, Kermanshah, Iran

**Keywords:** Trend, Incidence, Esophagus, Gastric, Colon, Cancer, Iran

## Abstract

**Background:**

Few studies have addressed the secular trend of malignancies in developing countries such as Iran. This study aimed to determine the trend in the incidence of gastrointestinal cancers during a period of 15 years in Kermanshah, Iran.

**Methods:**

All of the confirmed positive pathologic reports for esophageal, gastric and colorectal cancers from 1993 to 2007 were collected and compared with the data compiled in the provincial health center. The incidence rate was standardized for world population using a direct method. The Fay and Feuer method was used to calculate the 95% confidence intervals for each cancer in each year. Trends were investigated using linear regression.

**Results:**

Over the period, 2951 cases of gastrointestinal cancer were reported in Kermanshah Province. The age-standardized incidence rates for gastric, esophageal and colorectal cancers were 9.2, 8.1 and 4.5 per 100,000 respectively over 15 years. In all types, the incidence increased with age. The study showed that the incidence of gastric and esophageal cancers decreased annually by 0.28 (-0.67-0.11) and 0.36 (-0.70 – (-0.02)), respectively. Colorectal cancer demonstrated an increase in the incidence [0.14 (95% CI: -0.01-0.29) annually].

**Conclusion:**

A decrease in the incidence of gastric and esophageal cancers and an increase in the incidence of colorectal cancer are in line with reports from other developing countries in epidemiologic transition. Such trends warrant in depth investigation for the exact reasons.

## Introduction

Cancer is the leading cause of mortality in both developed and developing countries. Annually, it causes around seven million deaths (about 13% of all causes of death) in the world and is the second most common cause of death in developed countries and among the top three causes of adult death in developing countries.[1] Despite the predicted decrease in cancer related mortality rate by 2020, the number of cancer related deaths does not seem to decrease in future and it is estimated that the figure will even rise in 2020.[2] The major part of such rise occurs in developing counties where 70% of cancer related deaths will occur.[3] The highest number of cancer related deaths belongs to lung cancer (1.3 million deaths per year), gastric cancer (803000/year), colorectal cancer (639,000/year), liver cancer (610,000/year) and breast cancer (519,000). The epidemiologic pattern of cancer varies by sex; while for men, the most common cancers are lung, stomach, liver, colorectal, esophagus and prostate and in women are breast, lung, stomach, colorectal and cervical cancers.[4] Age-specific rate (ASR) in males in Fars Province for cancer of esophagus, stomach, small intestine, colon-rectum and liver was 1.05, 3.82, 0.15, 3.26 and 0.29 and these figures in females was 0.87, 1.60, 0.13, 2.41 and 0.42.[5] In southeastern Iran in Zahedan, for esophageal cancer, a higher prevalence of esophageal cancer was noticed in females, age group of older than 60 years and smokers and a most common type of squamous cell carcinoma.[6]

Such increase in number of cancers has many reasons. Undoubtedly, the increase in the number of new cases of cancer is due to the change in overall pattern of occurrence of diseases around the world. In other words, developing countries are experiencing an epidemiological transition period that is characterized by a gradual decrease in death due to communicable diseases and a parallel increase in death due to chronic conditions.[4] The major factors contributing to such changes are aging, life style change toward unhealthier patterns, decrease in incidence of infectious diseases and appropriate diagnosis and treatment of both communicable and non-communicable illnesses.

Among all cancers, malignancies of gastrointestinal tract are the most important ones in terms of a broad spectrum of cancers and overall incidence. While the middle East, including Iran, is considered as a low risk region for colorectal cancer, but gastric and esophageal cancers are still common dangers in some parts of Iran.[7] Considering the potential difference in the prevalence of risk factors of gastrointestinal malignancies in different provinces of Iran from one hand and recent improvement in health care services on the other, a study of secular trend in incidence of such malignancies could present a clear figure in epidemiology of gastrointestinal cancers over recent years. In absence of standard population-based cancer registries in most areas of Iran,[8] attempts to demonstrate the true incidence of cancer in different areas were mostly unsuccessful.[7][9][10][11][12][13] Yet, most studies have either not addressed the secular trend or not matched the rates to a standard population and are hence not comparable with international statistics.[9][10][11] In addition, there is no published report on trends in incidence of gastrointestinal cancers in Kemanshah Province (Western Iran). We aimed to provide a clear comparable figure of trends in incidence of gastrointestinal tract malignancies in Kermanshah Province, Western Iran.

## Materials and Methods

Kermanshah, the largest Province in the western part of Iran, is neighbor to Iraq. The estimated population of the province is around 2,000,000 people. Kermanshah has a pathology-based cancer registry system established in 2001. Upon approval from Ethics Committee of Kermanshah University of Medical Sciences and for the purpose of this study, all positive pathologic reports for a gastrointestinal cancer from 1993 to 2007 were included. The reports collected during 2001-2007 were compared with the computerized records of registered cancers and deaths filed in the statistical unit of the provincial health center. The registered cancers were coded using ICD-10. Esophageal, gastric and colorectal cancers were defined by C15, C16 and C18-C20, respectively. For every year that the data were available in the provincial registry, the registered cases were compared with pathologic reports and in case of a mismatch, the new cases were included. Data were then sorted by name, father’s name and age. All repeated cases were excluded.In addition, according to the address provided in pathology reports, the referred cases from neighboring provinces were excluded.

Descriptive statistics was used to fulfill the objectives of the study. STATA software (version 8) was implemented to determine the secular trend and other analyses. Age specific incidence rates were calculated for each cancer in each year by dividing the number of cancers diagnosed in each year by the population of Kermanshah at the same year. The population of Kermanshah was extracted from Statistical Center of Iran.[14] The population was divided into six age groups (0-39, 40-49, 50-59, 60-69, 70-79, ≥80 years). Age- standardized incidence rates were calculated using the World Standard Population.[15] Incidence rate was standardized using the direct method. The Fay and Feuer method was used to calculate the 95% confidence interval for standardized incidence rates.[16] The changes in the annual incidence of the cancers under study were calculated using simple linear regression.

## Results

A total of 1232 cases (336 women and 896 men) of gastric cancer were reported during the 15 years period of study in Kermanshah Province. The incidence was at a peak of 112 cases in 2007. Overall, the incidence of gastric cancer increased with age so that the age group ≥80 showed 3.2 times higher incidence than those in group 60-69 years. The standardized incidence during the 15-year period showed great fluctuations but overall, there was a decreasing trend from 10.6/100,000 (95%CI: 8.2-13.9) in 1993 to 9.1/100,000 (95%CI: 7.4-11.1) in 2007 (Table 1). The average decrease in the annual incidence of gastric cancer was 0.28 per 100,000 (95%CI: -0.67-(0.11)). The incidence was higher in men and the male to female ratio varied between 1.1 and 6.0. Overall, the standardized incidence rate of gastric cancer was 9.2 in 100,000.

Between 1993 and 2007, there were 1048 cases of reported esophageal cancer in Kermanshah Province of which 51% were men. As evidenced by the data, for most years, the incidence of this type of cancer increased with age (Table 2). Like gastric cancer, the incidence of esophageal cancer decreased during the period. The incidence decreased from 10, 7/100,000 (95%CI: 8.3-13.9) in 1993 to 5.0/100,000 (95%CI: 3.8-6.5) in 2007 (Table 2). The drop in the annual incidence of this cancer in Kermanshah Province was -0.36 per 100,000 (95%CI:-0.70 – (-0.02)). The standardized incidence rate of esophageal cancer was 8.1 per 100,000 over 15 years.

Among 665 cases of colorectal cancer reported during 1993 to 2007, 394 cases (59%) were men. More than half of the cases were reported between 2002 and 2007. Like gastric and esophageal cancers, the incidence of colorectal cancer increased with age in both genders (Table 3). Peak incidence rate for colorectal cancer belonged to year 2003 when it reached 6.1 (95%CI: 4.6-7.9) per 100,000 (Table 3). While erratic in terms of the annual incidence, it seems that colorectal cancer has an increasing incidence in Kermanshah Province. The average increase in annual incidence for colorectal cancer was 0.14 per 100,000 (95% CI: -0.01-(0.29)) (Table 4). In general, the incidence was higher in men with a maximum ratio of 2.9 compared to women. Over the 15 years of study, the standardized incidence of colorectal cancer was 4.5 per 100,000.

**Table 1:** Age-specific and -standardized rate/100,000 a of stomach cancer in Kermanshah Province

	**1993**	**1994**	**1995**	**1996**	**1997**	**1998**	**1999**	**2000**	**2001**	**2002**	**2003**	**2004**	**2005**	**2006**	**2007**
0-39	0.3	0.1	0.1	0.3	0.1	0.0	0.4	0.1	0.1	0.2	0.3	0.3	0.3	0.4	0.2
40-49	1.0	5.0	2.6	3.1	4.8	3.3	4.1	0.7	5.7	6.6	3.1	7.8	4.9	2.5	7.2
50-59	13.1	17.7	7.1	13.7	5.7	9.0	6.4	10.4	6.6	9.6	15.4	21.6	16.1	26.6	23.4
60-69	50.6	59.2	29.7	54.3	34.1	16.1	32.7	29.5	19.5	33.2	51.6	54.3	45.3	34.0	40.7
70-79	38.2	40.8	29.3	58.6	46.3	27.7	64.2	54.5	39.3	50.5	111.5	150.8	159.5	166.4	172.0
≥80	28.6	63.8	53.2	124.5	267.9	22.1	30.3	230.9	86.0	215.4	213.6	239.5	258.5	297.8	272.6
Age 95% CI	10.6 (8.2- 13.9)	13.8 (11.0- 17.4)	8.3 (6.0- 11.4)	15.8 (12.6-19.7)	15.5 (12.1-19.6)	5.1 (3.6 -7.2)	6.7 (5.1 -9.0)	8.7 (6.5 - 11.6)	7.3 (4.9- 10.7)	6.7 (5.1 - 8.7)	8.5 (6.8 - 10.6)	10.0 (8.2 - 12.1)	8.7 (7.1 - 10.8)	9.1 (7.4 - 11.1)	9.1 (7.4 - 11.0)

^a^ Rates have been standardized with world standard population

**Table 2:** Age-specific and -standardized rate/100,000 a of esophageal cancer in Kermanshah Province.

**Age (Year)**	**1993**	**1994**	**1995**	**1996**	**1997**	**<1998**	**1999**	**2000**	**2001**	**2002**	**2003**	**2004**	**2005**	**2006**	**2007**
0-39	0.0	0.1	0.0	0.1	0.1	0.1	0.4	0.1	0.1	0.1	0.0	0.1	0.0	0.2	0.1
40-49	2.9	4.0	2.1	5.2	2.6	1.1	2.7	0.7	5.7	3.7	7.6	6.2	4.0	2.5	1.8
50-59	12.3	18.6	10.7	20.9	8.5	5.0	11.5	11.7	9.3	16.5	32.1	24.3	32.2	25.3	18.9
60-69	53.6	40.9	35.4	54.3	39.6	20.1	32.7	22.9	29.3	52.1	43.5	71.9	55.1	23.7	27.6
70-79	42.0	38.8	25.1	52.3	67.3	23.1	51.9	32.0	19.6	47.1	79.1	107.1	109.1	117.7	83.9
≥80	22.8	15.9	44.3	124.5	228.3	22.1	91.1	461.8	103.1	305.1	485.3	196.0	211.5	160.4	247.8
Age a 95% CI	10.7 (8.3 - 13.9)	9.4 (7.3 - 12.2)	7.9 (5.7 - 10.9)	14.8 (11.7 - 18.6)	14.1 (11.0 - 18.0)	4.2 (2.8 - 6.2)	7.7 (5.7 - 10.2)	10.9 (8.2 - 14.3)	6.9 (4.6 - 10.2)	7.5 (5.7 - 9.6)	7.7 (6.0 - 9.7)	7.6 (6.0 - 9.5)	8.2 (6.5 - 10.1)	6.1 (4.7 - 7.7)	5.0 (3.8 - 6.5)

^a^ Rates have been standardized with world standard population

**Table 3:** Age-specific and -standardized rate/100,000 a of colorectal cancer in Kermanshah Province.

**Age (Year)**	**1993**	**1994**	**1995**	**1996**	**1997**	**1998**	**1999**	**2000**	**2001**	**2002**	**2003**	**2004**	**2005**	**2006**	**2007**
0-39	0.5	0.3	0.1	0.0	0.3	0.2	0.1	0.2	0.2	0.3	0.5	1.2	0.5	1.1	0.5
40-49	2.0	2.5	1.0	2.6	1.1	1.1	4.1	6.8	4.3	7.4	9.9	10.1	9.7	11.8	17.2
50-59	4.6	8.4	2.7	5.5	5.7	3.0	15.3	7.8	2.7	13.8	14.0	24.3	30.8	24.1	31.2
60-69	11.9	26.8	9.9	8.4	6.8	8.0	16.4	8.2	9.8	11.1	24.2	17.6	29.1	23.7	20.3
70-79	0	14.3	4.1	10.5	6.3	4.6	24.4	35.2	22.9	37.0	61.1	39.7	79.7	56.8	67.1
≥80	17.1	15.9	8.9	26.7	89.3	22.1	15.2	99.0	17.2	89.7	252.3	87.1	70.5	91.6	123.9
Age a 95% CI	3.5 (2.1 - 5.8)	5.2 (3.7 - 7.6)	3.1 (1.9 - 5.3)	4.5 (2.9 - 6.9)	4.6 (2.8 - 7.2)	2.0 (1.0 - 3.6)	4.4 (3.1 - 6.4)	5.8 (4.1 - 8.2)	2.8 (1.6 - 4.9)	3.6 (2.5 - 5.2)	6.1 (4.6 - 7.9)	4.9 (3.7 - 6.4)	6.0 (4.6 - 7.6)	5.6 (4.3 - 7.0)	5.7 (4.4 - 7.1)

^a^ Rates have been standardized with world standard population.

**Table 4:** Annual changes in incidence proportion

**Cancer**	**Annual changes in incidence/100000**	**95% confidence interval**
Gastric cancer	-0.28	-0.67-0.11
Esophageal cancer	-0.36	-0.70-(-0.02)
Colorectal cancer	0.14	-0.01-0.29

## Discussion

Based on the results of the study, while the figures for gastric and esophageal cancers were decreasing the incidence of colorectal cancer was increasing. The decreasing incidence of gastric cancer in Kermanshah Province seems to be in line with the decrease in the incidence and mortality of this cancer in most other countries (especially developed countries) which is considered an important finding.[17],18Although gastric cancer was the first cause of cancer related death in the world until the mid 90’s; the current mortality of this cancer is decreasing albeit more rapidly in developed world.[17][18]

For instance, in Australia, the mortality from gastric cancer decreased from 25.5 per 100,000 in 1950 to 6.7 per 100,000 in 1994. The highest incidence in 1996 belonged to Japan (65.0-95.0 per 100,000 in men and 27.3-40.1 per 100,000 in women), Korea (65.9 and 25.0 per 100,000 in men and women, respectively), and China (46.5 and 21.0 per 100,000 in men and women, respectively) and some Latin American countries. In fact, the difference in the incidence of gastric cancer among various countries is mainly related to the incidence of non-cardiac cancers since the incidence of cardiac cancers only varies from 1.5 to 4.5 per 100,000 around the world.[17][18]

According to an estimation by WHO, the incidence of stomach cancer in Iran (standardized by world standard population) was 26.1 and 11.1 per 100,000 in men and women, respectively in 2002.[19] However, reports from various geographical areas in Iran are different.[20] While northern and northwestern Iran are the areas with the highest incidence, Kerman in south of Iran has the lowest incidence- around 10.2 and 5.1 for men and women, respectively.13 In our study, the incidence rate for the last year reached to 12.1 and 5.5 per 100,000 mark in men and women,respectively which is of course lower than WHO estimates and similar to the report from Tehran.[12]

Since, the majority of studies in Iran are limited to a hospital or region where they did not calculate the incidence rate, the findings of our study cannot be easily compared to them.[5][6][9][10] There are studies that have investigated the secular trend but they are even more limited. Using morbidity odds ratios, Yazdizadeh et al. (2007) concluded that the incidence of gastric cancer increased in Fars and Tehran provinces during the past 30 years.21 The possible explanation for such increase might be partly attributed to difference in the method of investigating the secular trends and completeness of cancer case collection. In addition, two studies investigated the trends in two different time periods.

The reason for a decreased incidence in gastric cancer in Kermanshah may be related to the decrease in prevalence of H. pylori infection as well as higher consumption of fresh fruits and vegetables.[10][20] Improvement in health condition and sanitation as well as better treatment have reduced the chance of infection with H. pylori worldwide.[10][20][22] In addition, more effective treatment of other gastrointestinal disorders such as reflux, that plays a role in cardiac cancers, can contribute to the decrease of incidence of gastric cancer. In fact, the 37% increase in population of people aged 60 and over (the population at higher risk) in Kermanshah Province [14] makes the observed decrease even more important.

Esophageal cancer is reported as the sixth frequent cancer in the world.[23] It is characterized by rapid growth and high fatality. The decreasing trend in the incidence of this cancer is in line with other studies. [21][23][24][25][26] Esophageal cancer is decreasing in most areas of the world. However, reports indicate that the incidence of squamous cell carcinoma is decreasing while adenocarcinoma is increasing.[6][27] As an example, while esophageal cancer is an uncommon entity in the United States, there has been a 350% increase in the incidence of adenocarcinoma between 70’s and 90’s in men. A similar upward trend has also been noticed in many other developed countries.[27]

The highest incidence of esophageal cancer is reported in countries such as China, Mongolia, Ethiopia and Iran with the first three countries as high as 28.1 per 100,000 in.[23] In Iran, the highest incidence is reported in the northern region and in Golestan Province (among Turkmen tribe). In Mazandaran and Guilan provinces, only 300 Km to the west, the incidence drops sharply.[11] In a recent report from Fars Province the incidence of esophageal cancer in people over 15 was reported at 2.95 per 100,000. The incidence was decreased by 64% between 1977 and 1999 of the study period.[10] Similarly, in our report the incidence of esophageal cancer has significantly decreased over 15 years and standardized rate with the world population was 8.1 per 100,000. These findings are consistence with WHO world wide cancer reports but slightly higher than recent report from Tehran, Iran.[12][19]

Colorectal cancer is one of the most common malignancies in the western countries. However, numerous reports from Asia and Latin America indicate that its incidence is rising in these countries.[28] Kermanshah Province presents the same increasing incidence pattern of colorectal cancer reported from other Asian and Latin American countries and also from Fars Province in south of Iran.[7][27] While developed countries have high incidence of colorectal cancer, the increasing pattern in countries like Iran is alarming.

In the report from Fars Province, the age standardized rate for colon cancer among men was 1.61 per 100,000 during 1970-80 and 4.2 per 100,000 during 1990-2000 and there was a significant annual increase of 0.13 per 100,000 (p>0.05).[7] In Kermanshah, the incidence during 15 years of study was 4.5 per 100,000 with an annual increase of 0.14 per 100,000 on average. The International Agency for Research on Cancer (IRAC) estimates that the incidence of colorectal cancer in Iran will be 8.3 and 6.5 for men and women, respectively by 2020. This estimation and report from Tehran population-based survey (6.7 and 4.3/100,000 for colon and rectum, respectively) seems to be higher than ours.[12][29][30] Although this study was not designed to clearly identify the causes of such increase, it can be hypothesized that a change toward the western life style and eating unhealthy diet may be a major contributing factor.[28]

The current work is one of the few populationbased studies in Iran that addresses the trend in the incidence of gastrointestinal cancers. Studies on the secular trend of diseases are important in any country since they reflect the changes in the epidemiologic feature of the disease. These studies are more valuable and more difficult to conduct in developing countries where cancer registries are not population-based. Although the present study does not provide an ideally accurate image of incidence of gastrointestinal malignancies, the figures on trends in incidence provide valuable information. The major limiting factors that may have led to underestimation of the incidence are failure in detection of all cases, loss of some of the pathology reports, and referring the patients to other provinces mainly capital city of Tehran in the initial years of the study. Such factors can underestimate any decreases in secular trend. Likewise, any increase in incidence needs to be interpreted with caution. In addition, a well organized population-based cancer registry must be completed using other valuable sources such as medical and hospital records as well as pathology reports and death certificates though they are not easily available and linked to cancer registries in developing countries. Studies like ours may not reflect the true incidence for each year. However, even in population-based studies where cancer detection is based on regular and periodic screening programs, it is still not possible to claim that the true incidence has been calculated. This study is valuable in that it was able to report standardized rates and trends. Hence it can be compared with similar studies from other countries. In order to compare to similar reports from elsewhere, we used ICD coding system which classifies cancers according to the location of organ involvement instead of histology type of malignancy. [21][24][25]

A decrease in the incidence of gastric and esophageal cancer plus an increase in the incidence of colorectal cancer are all findings that warrant further investigation. These changes are compatible with an epidemiologic transition period where incidence of communicable diseases decreases and in turn incidence of the diseases that reflect a change in the life style toward a more western model increases. Attempts should be made to investigate about contributing factors to observed trends.

## References

[1] World Health Organization, International union against cancer. (2005). Global action against cancer.

[2] Mathers CD, Loncar D (2006). Projections of global mortality and burden of disease from 2002 to 2030. PLoS Med.

[3] Murray CJL, Lopez AD The global burden of disease: Harvard School of Public Health, World Bank, 1996.

[4] World Health Organization (2002). The World Health Report 2002.

[5] Mehrabani D, Tabei SZ, Heydari ST, Shamsina SJ, Shokrpour N, Amini M, Masoumi SJ, Julaee H, Farahmand M, Manafi A (2008). Cancer occurrence in Fars province, Southern Iran. Iran Red Crescent Med J.

[6] Ziaian B, MontazerIi V, Khazaiee R, Amini S, Karimi M, Mehrabani D (2010). Esophageal cancer occurrence in Southeastern Iran. J Res Med Sci.

[7] Hosseini SV, Izadpanah A, Yarmohammadi H (2004). Epidemiological changes in colorectal cancer in Shiraz, Iran: 1980-2000. ANZ J Surg.

[8] Etemadi A, Sadjadi A, Semnani S, Nouraie SM, Khademi H, Bahadori M (2008). Cancer registry in Iran: a brief overview. Arch Iran Med.

[9] Bafandeh Y, Farhang S (2009). Subsite distribution of gastric cancer in an area of high prevalence--northwest Iran. J Epidemiol.

[10] Bagheri Lankarani K, Mowla AF, Tabei SZ, Panjehshahin MR (2002). Changing epidemiology of esophageal cancer in Fars province, Iran. Iran J Med Sci.

[11] Islami F, Kamangar F, Aghcheli K, Fahimi S, Semnani S, Taghavi N, Marjani HA, Merat S, Nasseri-Moghaddam S, Pourshams A, Nouraie M, Khatibian M, Abedi B, Brazandeh MH, Ghaziani R, Sotoudeh M, Dawsey SM, Abnet CC, Taylor PR, Malekzadeh R (2004). Epidemiologic feartures of upper gastrointestinal tract cancers in Northeastern Iran. Br J Cancer.

[12] Mohagheghi MA, Mosavi-Jarrahi A, Malekzadeh R, Parkin M (2009). Cancer incidence in Tehran metropolis: the first report from the Tehran Population- based Cancer Registry, 1998- 2001. Arch Iran Med.

[13] Sadiadi A, Zahedi MJ, Moghadam SD, Nouraie M, Alimohammadian M, Ghorbani A (2007). The first populationbased cancer survey in Kerman province of Iran. Iranian Journal of Public Health.

[14] Statistical centre of Iran Estimation of population in Iran by province and year: Statistical centre of Iran, 2010.

[15] Segi M Cancer mortality for selected sites in 24 countries (1950-57). Sendari, Japan: Department of Public Health, Tohoku University of Medicine, 1960.

[16] Fay MP, Feuer EJ (1997). Confidence intervals for directly adjusted rates: a method based on the gamma distribution. Stat Med.

[17] Bertuccio P, Chatenoud L, Levi F, Praud D, Ferlay J, Negri E, Malvezzi M, La vecchia C (2009). Recent patterns in gastric cancer: A global overview. Int J Cancer.

[18] Kelley JR, Duggan JM (2003). Gastric cancer epidemiology and risk factors. J Clin Epidemiol.

[19] Ferlay J, Bray P, Fisani P, Parkin DM (2004). GLOBOCAN 2002: Cancer Incidence, Mortality and Prevalence Worldwide IARC CancerBase No. 5. Version 2.0..

[20] Malekzadeh R, Derakhshan MH, Malekzadeh Z (2009). Gastric cancer in Iran: epidemiology and risk factors. Arch Iran Med.

[21] Yazdizadeh B, Jarrahi AM, Mortazavi H, Mohagheghi MA, Tahmasebi S, Nahvijo A (2005). Time trends in the occurrence of major GI cancers in Iran. Asian Pac J Cancer Prev.

[22] Torres J, Lopez L, Lazcano E, Camorlinga M, Flores L, Munoz O (2005). Trends in Helicobacter pylori infection and gastric cancer in Mexico. Cancer Epidemiol Biomarkers Prev.

[23] Kollarova H, Machova L, Horakova D, Janoutova G, Janout V (2007). Epidemiology of esophageal cancer--an overview article. Biomed Pap Med Fac Univ Palacky Olomouc Czech Repub.

[24] Yeole BB (2008). Trends in cancer incidence in esophagus, stomach, colon, rectum and liver in males in India. Asian Pac J Cancer Prev.

[25] He YT, Hou J, Chen ZF, Qiao CY, Song GH, Meng FS, Jin HX, Chen C (2008). Trends in incidence of esophageal and gastric cardia cancer in high-risk areas in China. Eur J Cancer Prev.

[26] Semnani S, Sadjadi A, Fahimi S, Nouraie M, Naeimi M, Kabir J, Fakheri H, Saadatnia H, Ghavamnasiri MR, Malekzadeh R (2006). Declining incidence of esophageal cancer in the Turkmen Plain, eastern part of the Caspian Littoral of Iran: a retrospective cancer surveillance. Cancer Detect Prev.

[27] Lagergren J (2006). Etiology and risk factors for esophageal adenocarcinoma: possibilities for chemoprophylaxis?. Best Pract Res Clin Gastroenterol.

[28] Boyle P, Elena Leon M (2002). Epidemiology of colorectal cancer. Br Med Bull.

[29] International Agency for Research on Cancer. (2009). CANCER Mondial: International Agency for Research on Cancer.

[30] International Agency for Research on Cancer (2009). Globocan 2002 database: summary table by population.

